# Effectiveness of ultrasonography-guided caudal epidural steroid injection compared to the fluoroscopic application

**DOI:** 10.55730/1300-0144.5635

**Published:** 2023-04-04

**Authors:** Emrullah Cem KESİLMEZ, Kasım Zafer YÜKSEL, Ayşe Azak BOZAN, Cengizhan YAVUZ

**Affiliations:** 1 Department of Neurosurgery, Faculty of Medicine, Kahramanmaraş Sütçü İmam University, Kahramanmaraş, Turkey; 2 Department of Anesthesiology and Reanimation, Necip Fazıl State Hospital, Kahramanmaraş, Turkey; 3 Department of Anesthesiology and Reanimation, Faculty of Medicine, Kahramanmaraş Sütçü İmam University, Kahramanmaraş, Turkey

**Keywords:** Caudal epidural steroid injection, lower back pain, ultrasonography, fluoroscopy

## Abstract

**Background/aim:**

Caudal epidural steroid injection (CESI) has been increasingly used for treating lower back pain. However, there is still significant controversy about the efficacy and safety of different imaging techniques used to guide CESI. In this context, the objective of this study is to compare the efficacies of fluoroscopy- and ultrasonography-guided CESI in patients with chronic lower back pain.

**Materials and methods:**

The population of this retrospective, observational study consisted of all consecutive patients who underwent CESI for lower back pain between 2018 and 2020. Of the 371 patients included in the study sample, 192 had undergone fluoroscopy-guided CESI (Group F) and 179 ultrasonography-guided CESI (Group U). Patients’ pain and functional statuses were evaluated using the visual analog scale (VAS) and Oswestry Disability Index (ODI) immediately before (baseline) and after the procedure (postintervention day 0-D0), during the second week (D15), the first month (D30), and the third month (D90) after the procedure.

**Results:**

The mean age of Group F was significantly higher than that of Group U (p < 0.001). The number of patients with lumbar disc hernia was significantly higher in Group U, whereas the number of patients with spinal stenosis and lumbar disc hernia + spinal/lumbar stenosis was significantly higher in Group F (p = 0.001). The baseline and D0 ODI scores were significantly lower in Group U than in Group F (p = 0.006 and p = 0.017, respectively). There was no significant difference between the groups in other VAS and ODI scores (p > 0.05). Intragroup analyses revealed significant reductions in VAS and ODI scores over the follow-up period till D30 compared to the baseline scores in each group (p < 0.001). The decrease recorded in the ODI score between the D15 and baseline measurements was significantly higher in Group F than in Group U (p = 0.006).

**Conclusion:**

The study findings indicated that ultrasound-guided CESI was as effective as fluoroscopy-guided CESI in treating chronic lower back pain.

## 1. Introduction

Chronic lower back and leg pain are widespread problems that adversely affect the quality of life. The most common causes of chronic lower back pain and leg pain are lumbar discopathies, including lumbar disc herniation and spinal stenosis (narrow canal) [1–4].

Various conservative treatment strategies, including resting, oral medication, physical therapy, and lifestyle modifications, are recommended to overcome lower back and leg pain [5,6]. In addition, interventional pain management strategies are recommended in patients with persistent lower back and leg pain. Among these strategies, epidural steroid injection (ESI) stands out as the most common nonsurgical method for treating lower back pain and leg pain caused by lumbar discopathy and spinal stenosis [4,7–10].

Accurate localization of the sacral hiatus is the most critical aspect of an injection, regardless of the approach being used [5,11–13]. The most common ESI approaches are transforaminal anterior, interlaminar, and caudal injections [6]. The caudal ESI (CESI) has been preferred over other approaches considering its ease of administration and the hemorrhagic complications associated with different approaches [6,14,15]. Several methods have been resorted to in localizing the sacral hiatus, including palpation of the posterior superior iliac crest and imaging techniques such as fluoroscopy and ultrasound (US) [5].

However, given the difficulty in localizing the sacral hiatus and accurate insertion of the needle into the epidural space, palpation of the posterior superior iliac crest, as a blind technique, reportedly failed in up to 38.5% of the cases [15,16]. Alternatively, fluoroscopy-guided ESI, as a method that improves patients’ pain and functional statuses, has been regarded as the standard of care for conservative treatment [10,15]. Nevertheless, fluoroscopy-guided ESI also has some disadvantages, such as repeated exposure to ionizing radiation and contrast agent injection, regardless of the setting [10,17]. The studies on an alternative technique with comparable efficacy but without the drawbacks of fluoroscopy-guided ESI have reported promising efficacy and safety rates for US-guided CESI [6,10,17–20]. Besides, US-guided CESI reportedly offered additional advantages, including ease of application, simultaneous real-time image acquisition, availability of postlaminectomy cases, and absence of radiation [19,21,22]. Therefore, CESI has been increasingly used for pain management, yet there is still significant controversy around the efficacy and safety of different imaging techniques used to guide CESI [23].

In this context, this study was carried out to compare the advantages and disadvantages of fluoroscopy- and US-guided CESI in patients with chronic lower back pain.

## 2. Materials and methods

### 2.1. Study design

This retrospective, observational study was carried out in the Department of Neurosurgery, Kahramanmaraş Sütçü İmam University Medical Faculty Hospital, Maraş, Turkey, between 2018 and 2020. All procedures involving human participants were carried out in accordance with the ethical standards of the institutional and/or national research committee, the 1964 Declaration of Helsinki, and its later amendments or comparable ethical standards. The study protocol was approved by the Clinical Studies Bioethics Committee of the Medical Faculty of Kahramanmaraş Sütçü İmam University (Decision Date: March 8th, 2022; Session Number: 2022/09; Decision No: 03). The written consent could not be taken from the patients due to the retrospective design of the study and unanimity of data.

### 2.2. Population and sample

**Figure 1:**
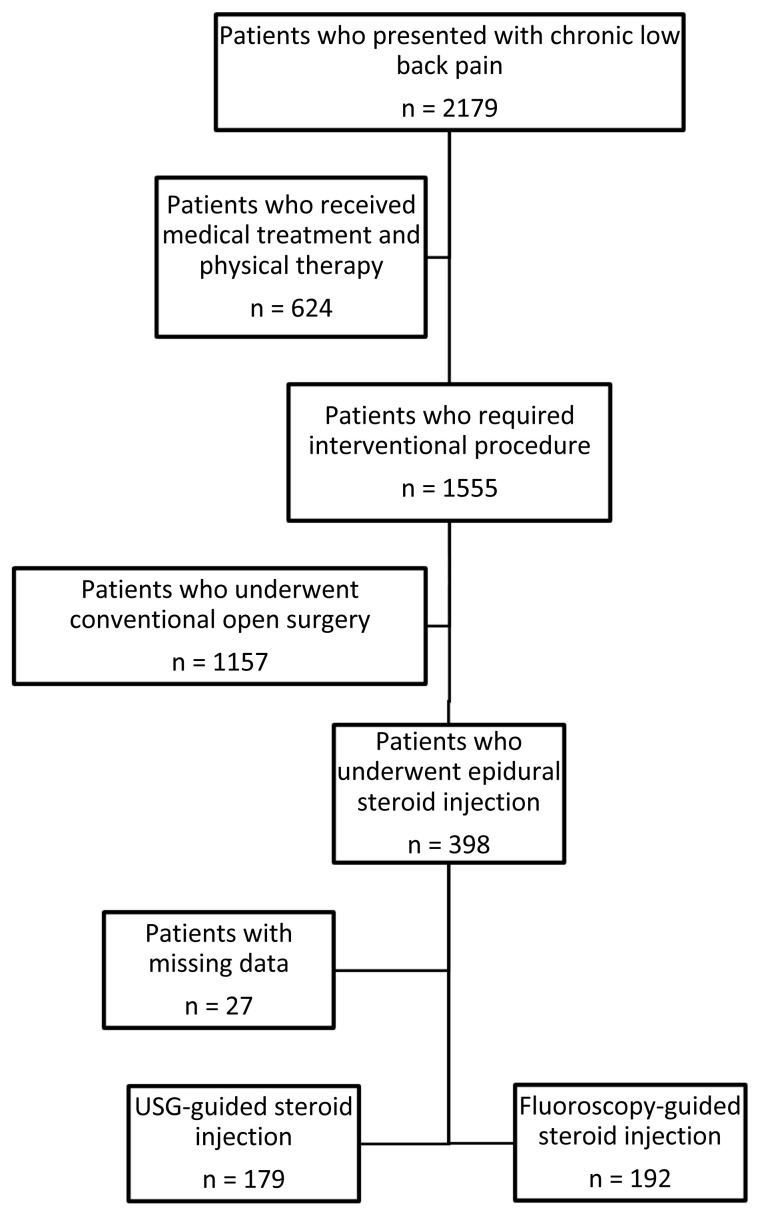
Study flow chart.

The study population consisted of all consecutive patients aged 18 years or above who underwent CESI for lower back pain in the Department of Neurosurgery, Kahramanmaraş Sütçü İmam University Medical Faculty Hospital, Kahramanmaraş, Turkey, between 2018 and 2020. The study inclusion criteria were having been diagnosed with facet joint hypertrophy, canal stenosis due to hypertrophy of ligamentum flavum, or disc hernia diagnosed using lumbar magnetic resonance imaging, having undergone fluoroscopy or US-guided CESI for chronic lower back pain after no improvement was obtained with conservative treatment, and having been followed up for at least three months. On the other hand, patients who underwent conventional open surgery for lumbar discopathies, patients with missing data, neurological deficits associated with the underlying pathology, cauda equina syndrome, chronic peripheral neuropathy, arterial vascular disease, degenerative spondylolisthesis, facet arthrosis, hip osteoarthritis, congenital or degenerative lumbar spinal stenosis, concurrent infections and malignancy, coagulopathy, allergy to iodinated contrast or medications, pregnancy, and pain for less than six weeks were excluded from the study (Figure 1). In the end, 371 patients were included in the study sample. Of these patients, 192 had undergone fluoroscopy-guided CESI (Group F) and 179 ultrasonography-guided CESI (Group U).

### 2.3. Data collection

As an institutional policy, patients were evaluated immediately before (baseline) and after (postintervention day 0-D0) the procedure, during the second week (D15), the first month (D30), and the third month (D90) after the procedure. The visual analog scale (VAS) [4] and Oswestry Disability Index (ODI) [24] were used to assess the clinical effectiveness of the interventional strategy and patients’ functional impairment [5]. The VAS and ODI scores were recorded into a predesigned worksheet. The Turkish validation of the ODI was completed previously [25].

The 100 mm-long standard VAS is a self-report scale scored between 0 (“no pain”) mm to 100 mm (“the worst pain”). The ODI involves administering a questionnaire that addresses the impairment in performing daily activities due to lower back pain. ODI consisted of ten items. Each item is assigned a score between 0 (“the least amount of disability”) and 5 (“the most severe disability”). These scores were added and then multiplied by two to obtain the overall ODI score [5].

### 2.4. Interventions

The CESI procedure was performed while the patients were in the prone position in the operating room by placing a cushion beneath the abdomen for optimal imaging. All CESI procedures were performed by experienced neurosurgeons (ECK, ZY). During the interventions, the patients were monitored via pulse oximetry, blood pressure, and electrocardiography. Intravenous (IV) midazolam and fentanyl were used to obtain sedative anesthesia.

The skin overlying the sacrococcygeal area was prepared using iodine-based antiseptic solutions followed by local anesthesia application with 4 mL of prilocaine diluted in 10 mL of saline solution. A 20-gauge, 90-mm spinal needle with its stylet was inserted just below the sacral hiatus at a 45° angle till the bony tissue. The needle was slightly withdrawn and then advanced further to enter the sacral canal.

A monoplanar fluoroscope (Siremobil Compact L, C-arm, Siemens Healthineers, Erlangen, Germany) was used to perform fluoroscopy-guided CESI. The position of the needle in the caudal epidural space was confirmed via a lateral fluoroscopic view. After negative aspiration, 1 mL of contrast medium (iohexol, 300 mg iodine per mL) was injected. Antero-posterior and lateral radiographic views were used to exclude intravascular, intrathecal, and/or soft tissue infiltration [4].

A linear 9 MHz probe (Aloka Prosound Alpha-7, Hitachi Aloka Medical Systems, Tokyo, Japan) was used to perform US-guided CESI. The position of the needle was confirmed by the probe’s longitudinal placement to the vertebral axis [5].

A mixture of 8 mL 0.5% bupivacaine hydrochloride (40 mg), 2 mL dexamethasone (4 mg/mL), and 10 mL normal saline was used for epidural injections. This mixture was slowly injected into the epidural space at a rate of 10 mL/min. The patients were discharged after they were allowed to recover in the postanesthesia care unit for one hour [4,5].

### 2.5. Statistical analysis

The descriptive statistics obtained from the collected data were expressed as mean ± standard deviation and median with minimum–maximum values or interquartile ranges values in the case of continuous variables with and without normal distribution, respectively. Additionally, categorical variables were expressed as numbers and percentage values. The Shapiro–Wilk, Kolmogorov–Smirnov, and Anderson–Darling tests were used to analyze the normal distribution characteristics of the numerical variables.

The independent samples t-test and the Mann–Whitney U test were used to compare two independent groups where numerical variables conformed (age) and did not conform (VAS and ODI scores) to the normal distribution, respectively.

Pearson’s chi-squared test was used to compare the differences between categorical variables (sex and diagnosis) in 2 × 2 tables.

The Kruskal–Wallis test was used to compare more than two independent groups in terms of diagnoses where numerical variables did not conform to the normal distribution. In addition, the Dwass–Steel–Critchlow–Fligner test was used to evaluate the differences between the groups in terms of diagnoses.

The Friedman test was used to analyze the changes in VAS and ODI scores over the study period. The Durbin–Conover test was used to perform multiple comparisons in order to find significant pairwise relationships.

Jamovi project 2.3.24 (Jamovi, version 2.3.24, 2023, retrieved from https://www.jamovi.org) and JASP 0.17.1 (Jeffreys’ Amazing Statistics Program, version 0.16.1, 2023, retrieved from https://jasp-stats.org) software packages were used in the statistical analyses. The probability (p) statistics of ≤0.05 were deemed to indicate statistical significance.

## 3. Results

The distribution of demographic and clinical characteristics by the groups is given in Table 1. The mean age of the patients in Group F was significantly higher than in Group U (59.3 ± 12.2 vs. 55.1 ± 11.0 years, p < 0.001). There was no significant difference between the groups in terms of sex distribution and smoking status (p = 0.847 and p = 0.472, respectively). There were significant differences between the groups in educational status and diagnoses (p = 0.0011 and p = 0.001, respectively). In addition, the number of patients with lumbar disc hernia was significantly higher in Group U, whereas the number of patients with spinal stenosis and lumbar disc hernia + spinal/lumbar stenosis was significantly higher in Group F (Table 1). There were no significant differences between the groups in terms of laboratory parameters (p > 0.05) (Table 2).

There was also no significant difference between the groups in terms of VAS scores assessed at different endpoints (p > 0.05 for all cases) (Table 3). On the other hand, the baseline and D0 ODI scores were significantly lower in Group U than in Group F (p = 0.006 and p = 0.017, respectively). There was no significant difference between the groups in ODI scores assessed at other endpoints (p > 0.05).

Intragroup analyses revealed significant reductions in VAS and ODI scores over the follow-up period till D30 compared to the baseline scores in each group (p < 0.001) (Table 3).

**Figure 2:**
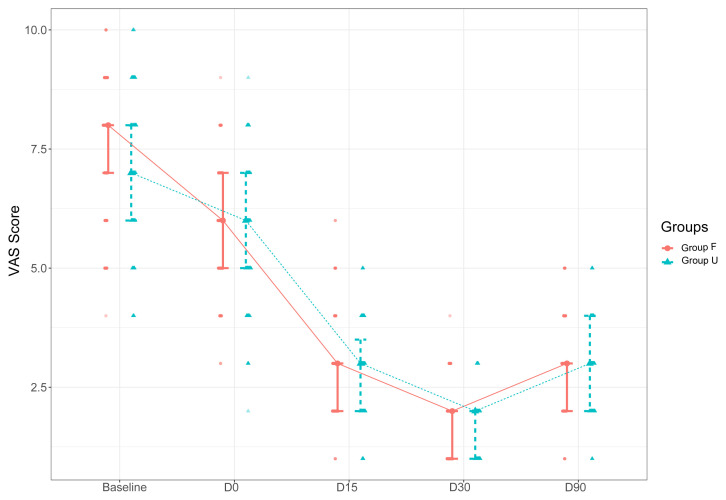
Changes in the VAS scores of the patients in Groups F and U over the study period (VAS: visual analog scale, CESI: caudal epidural steroid injection, Group F: fluoroscopy-guided CESI, US: ultrasound, Group U: US-guided CESI, D: postintervention day).

**Figure 3:**
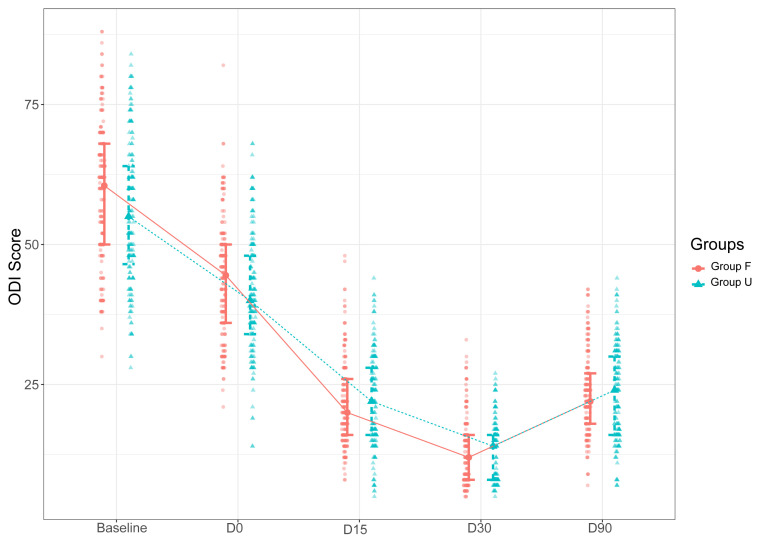
Changes in the ODI scores of the patients in Groups F and U over the study period (ODI: Oswestry Disability Index, CESI: caudal epidural steroid injection, Group F: fluoroscopy-guided CESI, US: ultrasound, Group U: US-guided CESI, D: postintervention day).

The D90 VAS and ODI scores were significantly higher than D30 VAS and ODI scores (Figures 2 and 3).

The percent (%) changes in VAS and ODI scores recorded between different endpoints and at the baseline are summarized in Table 4. There was no significant difference between the groups in the percent changes in VAS scores over the study period (p > 0.05).

There was no significant difference between the groups in the percent changes in ODI scores assessed over the study period compared to the baseline values (p > 0.05) (Table 4), except for the decrease recorded in the ODI score between the D15 and baseline measurements, which was significantly higher in Group F than in Group U (p = 0.006).

There was also no significant difference between the groups created based on different diagnoses in the percent changes of the VAS and ODI values assessed over the study period compared to the baseline values (p > 0.05) (Table 5), except for the change recorded in the ODI score between the D30 and baseline measurements, which was significantly higher in patients with spinal stenosis than those with the combined pathology (lumbar disc hernia + stenosis) (p = 0.023).

## 4. Discussion

The study findings, including the improved pain (VAS) and clinical assessment (ODI) scores assessed during the pre- and postinterventional acute and chronic follow-up periods up until 3 months after the intervention indicated that US-guided CESI was as effective as fluoroscopy-guided CESI in treating chronic lower back pain.

Invasive interventional techniques are recommended in cases where conventional and conservative techniques fall short in achieving functional recovery in patients with chronic lower back pain. Among these techniques, CESI has gained popularity given its feasibility and safety for treating chronic lower back pain [26]. Corticosteroids reduce pain with their anti-inflammatory effects and membrane-stabilizing properties in the ganglia of the dorsal roots and also suppress ectopic stimuli in damaged nerve fibers [14,27–29]. Based on these underlying action mechanisms, steroids were preferred for the caudal epidural injections in this study.

Several studies have comparatively addressed the two most frequently used CESI techniques, i.e. fluoroscopy- or US-guided CESI [4,10,14,18,23,30]. Each of these studies reported different results on the advantages or disadvantages of each technique in terms of technical considerations, complications, and financial aspects [4,5,31–34]. The discrepancies between these studies might be attributed to methodological heterogeneities [14]. Nevertheless, there is still significant controversy around the efficacy and safety of these imaging techniques used to guide CESI. In a randomized clinical trial, Poutoglidou et al. [5] found that all the blind technique and fluoroscopy- and US-guided CESI techniques led to significant improvements in the VAS and ODI scores assessed one month after the intervention compared to the baseline values, yet did not find any significant difference between the blind technique and fluoroscopy- and US-guided CESI in terms of pain relief and functional improvement. On the other hand, Senkal et al. [6] found that US-guided CESI was superior to fluoroscopy-guided CESI in terms of successful injection rate on the first attempt and duration of intervention, yet both were comparable in terms of Numeric Rating Scale and ODI scores. Several other studies have found the clinical efficacies of fluoroscopy- and US-guided CESI comparable [15,17,20,35]. Similarly, Akkaya et al. [19] reported that the fluoroscopy- and US-guided CESI resulted in comparable outcomes in postlaminectomy patients. In comparison, the technical success rate and the duration of intervention were not investigated in this study. On the other hand, the findings of this study on the degree of improvement in pain and clinical functions were in line with the majority of the literature data, that is, both fluoroscopy- and US-guided CESI techniques resulted in comparable clinical efficacies, yet US-guided CESI was superior to fluoroscopy-guided CESI considering the absence of radiation exposure.

The self-reported questionnaires are the most widely used common data collection tools for evaluating the functional limitations in patients with chronic lower back pain. Among these tools, VAS assesses pain intensity [4,5,10,17,19,23], whereas ODI assesses patients’ ability to manage daily life [5,6,17,19,20].

Some studies stratified the outcomes of different CESI techniques according to the type of disc pathology [6,20] and studies that featured radiculopathy as an inclusion criterion depending on the methodology [17,23]. In addition, other studies analyzed the postinterventional outcomes of different CESI techniques in patients only with lumbar disc hernia or spinal stenosis, contrary to the studies that included patients with varying disc pathologies, including spondyloses [4,5,10,14,15,20]. Nevertheless, only a limited number of studies evaluated the outcomes of different CESI techniques in treating chronic lower back pain [3,36]. These studies reported that injections were more effective in radiculopathies caused by disc hernia than spinal stenosis. All these studies reported significant improvements with the use of CESI in almost all patient groups regardless of the technique used. In comparison, in this study, the pain scores and the degree of clinical improvement were compared between patients with three different disc pathology groups. Consequently, no significant change was observed between the groups created based on different disc pathologies in the percent changes recorded in VAS and ODI scores during the study period compared to the baseline values. This finding does not contradict the relevant findings reported in the literature in that most patients benefit from these interventional treatment modalities regardless of the technique used, in terms of improvement in pain scores and performing daily clinical activities.

## 5. Limitations of the study

In addition to being the first study on the subject and the large sample size, which may be deemaed the study’s primary strength, there were also some limitations to the study, the most important being its retrospective design. Secondly, the lack of randomization in selecting the CESI technique and significant differences between the patient groups in demographic and clinical characteristics may be deemed other limitations of the study. Thirdly, the 3-month follow-up period might be insufficient to evaluate the long-term outcomes of different CESI techniques.

## 6. Conclusion

The study findings indicated that US-guided CESI was as effective as fluoroscopy-guided CESI in treating chronic lower back pain. In addition, the fact that patients undergoing US-guided CESI are not exposed to radiation renders it superior to fluoroscopy-guided CESI.

## Figures and Tables

**Table 1:** Demographic and clinical data of the patients in Groups F and U.

	Group		
	Group F (fluoroscopy-guided CESI) (n = 192)	Group U (US-guided CESI) (n = 179)	p-value
**Sex** ‡			
Female	114 (59.4)	109 (60.9)	0.847*
Male	78 (40.6)	70 (39.1)	
**Age (***year***)** †	59.3 ± 12.2	55.1 ± 11.0	**<0.001** **
**Smoking** ‡	94 (49.0)	80 (44.7)	0.472*
**Education** ‡			
Illiterate	13 (6.8) a	6 (3.4) a	**0.001** *
Primary	47 (24.5) a	19 (10.6) b	
Secondary	10 (5.2) a	23 (12.8) b	
College	98 (51.0) a	107 (59.8) a	
University	24 (12.5) a	24 (13.4) a	
**Diagnosis** ‡			
LDH	89 (46.4) a	118 (65.9) b	**0.001** *
Spinal stenosis	50 (26.0) a	28 (15.6) b	
Combined (LDH + spinal/lumbar stenosis)	53 (27.6) a	33 (18.4) b	

CESI: caudal epidural steroid injection, US: ultrasound.

a,b Different letters show the statistical significance in each row.

‡ n (%),

† mean ± standard deviation.

* Pearson chi-squared test.

** Independent samples t-test.

**Table 2:** Laboratory investigations of the patients in the study groups.

	Group		
	Group F (Fluoroscopy-guided CESI) (n = 192)	Group U (US-guided CESI) (n = 179)	p-value
**Hemoglobin (g/dL)** †	13.0 ± 0.7	13.0 ± 0.7	0.949
**Leucocyte count (×10^9^/L)** †	5.9 ± 0.8	5.8 ± 0.9	0.470
**Platelet count (×10^9^/L)** †	302.9 ± 63.6	298.7 ± 63.7	0.521
Sodium (mEq/L) †	137.6 ± 3.1	137.8 ± 2.8	0.451
Potassium (mEq/L) †	4.3 ± 0.5	4.3 ± 0.5	0.449
**Blood urea nitrogen (mg/dL)** †	12.3 ± 3.1	12.2 ± 3.0	0.793
Creatinine (mg/d/L) †	0.6 ± 0.1	0.6 ± 0.1	0.822
**Aspartate aminotransferase (IU/L)** †	19.7 ± 5.9	20.0 ± 5.7	0.634
**Alanine aminotransferase (IU/L)** †	19.6 ± 5.6	20.5 ± 6.0	0.165

† mean ± standard deviation.

CESI: caudal epidural steroid injection, US: ultrasound.

Independent samples t-test.

**Table 3:** VAS and ODI scores of the patients in Groups F and U.

	Groups		
	Group F (Fluoroscopy-guided CESI) (n = 192)	Group U (US-guided CESI) (n = 179)	p-value*
**VAS Scores** §			
Baseline	8.0 [4.0–10.0]	7.0 [4.0–10.0]	0.083
D0	6.0 [3.0–9.0]	6.0 [2.0–9.0]	0.112
D15	3.0 [1.0–6.0]	3.0 [1.0–5.0]	0.643
D30	2.0 [1.0–4.0]	2.0 [1.0–3.0]	0.648
D90	3.0 [1.0–5.0]	3.0 [1.0–5.0]	0.417
*p-value* **	***p < 0.001***	***p < 0.001***	
**ODI scores** §			
Baseline	60.5 [30.0–88.0]	55.0 [28.0–84.0]	**0.006**
D0	44.5 [21.0–82.0]	40.0 [14.0–68.0]	**0.017**
D15	20.0 [8.0–48.0]	22.0 [5.0–44.0]	0.402
D30	12.0 [5.0–33.0]	14.0 [5.0–27.0]	0.893
D90	22.0 [7.0–42.0]	24.0 [7.0–44.0]	0.736
*p-value* **	***p < 0.001***	***p < 0.001***	

CESI: caudal epidural steroid injection, US: ultrasound, VAS: visual analog score; ODI: Oswestry Disability Index, D: postintervention day.

§ median [min–max].

* Mann–Whitney U test.

** Friedman test.

**Table 4:** Comparison of the groups according to the percent changes (%) in VAS and ODI scores in different evaluation periods from the baseline measurements.

		Group		
Baseline	Evaluation time	Group F (Fluoroscopy-guided CESI) (n = 192)	Group U (US-guided CESI) (n = 179)	p-value
**VAS** §	Δ VAS D0 (%)	−20.0 [−55.6 to 20.0]	−20.0 [−57.1 to 0.0]	0.894
	Δ VAS D15 (%)	−62.5 [−87.5 to −16.7]	−60.0 [−87.5 to −28.6]	0.058
	Δ VAS D30 (%)	−77.8 [−90.0 to −50.0]	−77.8 [−88.9 to −40.0]	0.541
	Δ VAS D90 (%)	−62.5 [−88.9 to −16.7]	−60.0 [−87.5 to −28.6]	0.380
**ODI** §	Δ ODI D0 (%)	−25.0 [−61.5 to 6.9]	−25.4 [−58.8 to 3.3]	0.672
	Δ ODI D15 (%)	−64.9 [−86.8 to −13.6]	−60.0 [−90.0 to −29.2]	**0.006**
	Δ ODI D30 (%)	−79.2 [−92.4 to −40.9]	−78.4 [−90.3 to −50.0]	0.127
	Δ ODI D90 (%)	−62.3 [−88.0 to −13.6]	−58.3 [−100.0 to −22.7]	0.128

CESI: caudal epidural steroid injection, US: ultrasound, VAS: visual analog score; ODI: Oswestry disability index, D: postintervention day.

§ median [min–max]

Mann–Whitney U test was used.

**Table 5:** Comparison of the groups according to the percent changes in VAS and ODI scores in different evaluation periods from the baseline measurements.

		Patients with			
Baseline	Evaluation time	Lumbar disc hernia (n = 207)	Spinal stenosis (n = 78)	Lumbar disc hernia +stenosis (n = 86)	p-value
**VAS**	Δ VAS D0 (%)	−16.7 [−50.0 to 20.0]	−22.2 [−57.1 to 0.0]	−22.2 [−55.6 to 16.7]	0.096
	Δ VAS D15 (%)	−62.5 [−87.5 to −28.6]	−62.5 [−77.8 to 25.0]	−62.5 [−87.5 to −16.7]	0.098
	Δ VAS D30 (%)	−77.8 [−88.9 to −40.0]	−75.0 [−90.0 to 50.0]	−80.0 [−88.9 to −50.0]	0.054
	Δ VAS D90 (%)	−60.0 [−87.5 to −28.6]	−60.0 [−77.8 to 37.5]	−60.0 [−88.9 to −16.7]	0.769
**ODI**	Δ VAS D0 (%)	−25.0 [−58.8 to 6.9]	−26.9 [−54.5 to 3.3]	−26.9 [−61.5 to 5.0]	0.368
	Δ VAS D15 (%)	−62.9 [−90.0 to −28.0]	−61.8 [−83.3 to 24.2]	−64.0 [−86.8 to −13.6]	0.345
	Δ VAS D30 (%)	−81.5 [−90.0 to −40.9]	−79.0 [−92.4 to 48.0]	−77.0 [−91.9 to −46.8]	**0.023**
	Δ VAS D90 (%)	−60.8 [−88.0 to −13.6]	−59.2 [−100.0 to 22.7]	−59.8 [−80.6 to −28.6]	0.757

VAS: visual analog score; ODI: Oswestry disability index, D: postintervention day.

§ median [min–max].

Kruskal–Wallis test was used.
